# m7G Methyltransferase METTL1 Promotes Post-ischemic Angiogenesis via Promoting VEGFA mRNA Translation

**DOI:** 10.3389/fcell.2021.642080

**Published:** 2021-05-31

**Authors:** Yongchao Zhao, Lingqiu Kong, Zhiqiang Pei, Fuhai Li, Chaofu Li, Xiaolei Sun, Bei Shi, Junbo Ge

**Affiliations:** ^1^Department of Cardiology, Affiliated Hospital of Zunyi Medical University, Zunyi, China; ^2^Department of Cardiology, Zhongshan Hospital, Fudan University, Shanghai, China; ^3^Shanghai Institute of Cardiovascular Diseases, Shanghai, China

**Keywords:** N7-methylguanosine, methyltransferase like 1, post-ischemic angiogenesis, peripheral artery disease, vascular endothelial growth factor A

## Abstract

Post-transcriptional modifications play pivotal roles in various pathological processes and ischemic disorders. However, the role of N7-methylguanosine (m7G), particularly m7G in mRNA, on post-ischemic angiogenesis remains largely unknown. Here, we identified that methyltransferase like 1 (METTL1) was a critical candidate responsible for a global decrease of m7G within mRNA from the ischemic tissues. The *in vivo* gene transfer of METTL1 improved blood flow recovery and increased angiogenesis with enhanced mRNA m7G upon post-ischemic injury. Increased METTL1 expression using plasmid transfection *in vitro* promoted HUVECs proliferation, migration, and tube formation with a global increase of m7G in mRNA. Mechanistically, METTL1 promoted VEGFA mRNA translation in an m7G methylation-dependent manner. Our findings emphasize a critical link between mRNA m7G and ischemia and provide a novel insight of targeting METTL1 in the therapeutic angiogenesis for ischemic disorders, including peripheral arterial disease.

## Introduction

Ischemic cardiovascular disease, including myocardial infarction and peripheral arterial disease (PAD), is the leading cause of mobility and mortality worldwide (Murabito et al., 2002). PAD typically presents as intermittent claudication but often progresses to even critical limb ischemia (CLI) characterized by pain, ulcers, and gangrene, leading to amputation and early death (Foley et al., 2016; Hess et al., 2017).

Vascular occlusion serves as the significant pathology of ischemic diseases. It is exacerbated by insufficient angiogenesis, which is essential for tissue regeneration or repair, muscle and bone repair, hypoxia, and chronic ischemia. Besides endovascular treatment, few therapeutic options are available for the blood flow recovery to ischemic tissues (Norgren et al., 2007; Gupta et al., 2009; Hess et al., 2017). Therefore, a better understanding to identify pivotal regulators for therapeutic targets to promote post-ischemic angiogenesis would be of great significance.

Epigenetic modifications in DNA methylation (Cooper and Keaney, 2011; Rao et al., 2011), histone acetylation (Yan et al., 2018; Fu et al., 2020), and N^6^-methyladenosine (m6A) (Frye et al., 2018; Mathiyalagan et al., 2019) have been illustrated to play essential roles in the therapeutic angiogenesis of post-ischemic disorders. Besides m6A, N7-methylguanosine (m7G) serves another positively charged modification is the ubiquitous in mRNA cap (Cowling, 2009; Furuichi, 2015), tRNA (Cartlidge et al., 2005), rRNA (Létoquart et al., 2014) and newly demonstrated to exist in internal mRNA (Zhang et al., 2019) which is installed by methyltransferase like 1 (METTL1) and WD repeat domain 4 (WDR4) complex in mammals (Guy and Phizicky, 2014; Lin et al., 2018). Function roles of m7G methylation within mRNA are associated with translation efficiency alteration of transcripts that contain METTL1 and WDR4 affected m7G sites (Guy and Phizicky, 2014; Malbec et al., 2019; Zhang et al., 2019). These internal m7G methylation impact RNA function and have been suggested associated with human diseases (Lin et al., 2018). Mouse embryonic stem cells lacking METTL1 or WDR4 display defects in self-renewal and neural differentiation (Lin et al., 2018). METTL1-mediated m7G methylation could also regulate the pluripotency and differentiation of human-induced pluripotent stem cells, thus suggesting its potential roles in vasculogenesis and the treatment of vascular diseases (Deng et al., 2020). Nevertheless, the evidence for crucial signaling between the relevant catalases linking m7G and angiogenesis remains unclear. The vascular endothelial growth factor (VEGF) family, particularly for VEGFA, is a critical angiogenic factor for angiogenesis to regulate the progression of ischemic diseases (Claesson-Welsh and Welsh, 2013; Sun et al., 2016; Siveen et al., 2017; Zou et al., 2019). The expression of VEGFA is influenced by histone methylation (Zhang et al., 2013; Ren, 2016), non-coding RNAs (Tan et al., 2018; Lu et al., 2020), and other epigenetic regulatory networks. However, information on the post-transcriptional modifications of m7G within VEGFA mRNA involved in post-ischemic angiogenesis has not been addressed.

In the present study, we reveal that the internal mRNA m7G methyltransferase METTL1, rather than WDR4, serves as a major responder upon post-ischemic insults and leads to a global decrease of m7G methylation within mRNA. Utilizing gains of function strategies, we identified that METTL1 improved blood flow recovery and increased angiogenesis with enhanced m7G upon post-ischemic injury *in vivo* and increased HUVECs angiogenesis *in vitro* via promoting the VEGFA mRNA translation in an m7G methylation-dependent manner. Our study uncovered a novel direct link between m7G methylation and post-ischemic angiogenesis, thus providing insight into targeting METTL1 in the treatment of ischemic disorders, including PAD.

## Materials and Methods

All animal studies were conducted according to the criteria of the “Guide for the Care and Use of Laboratory Animals” prepared by the National Academy of Sciences and published by the National Institutes of Health and approved by the Animal Care and Utilization Committee of Zunyi Medical University, China.

### Hind-Limb Ischemia

Eight-week-old C57B6LJ wild-type mice were anesthetized with 1.5–2% isoflurane. A small incision was made over the femoral vasculature, and the femoral artery was separated from the vein and nerve. The segment proximal to the outlet of the profundal femoris artery and distal to the outlet of the saphenous artery was ligated and then removed between the ligatures. The blood flow was measured by a Laser Doppler ultrasound scanning system (PeriScan PIM 3 system, Perimed, Sweden) before, immediately after, and at 3, 7, 14, and 21 days after femoral arteriotomy. The perfusion ratio of the ischemic to non-ischemic was quantified for each animal at each time point. In this study, adeno-associated virus (AAV) serotype 9 (Oobio, Shanghai, China) was used for sustained overexpression of *METTL1* gene under biosafety level 2 conditions. In brief, 1 × 10^11^ plaque-forming units (PFUs) control AAV (OE-Ctrl) and overexpression AAV (OE-METTL1) diluted in 10ul PBS were injected into the 4-week-old mice gastrocnemius before surgery. Four weeks later, the mice were subjected to hind-limb ischemia as described above. Twenty-one days after femoral arteriotomy, the gastrocnemius was collected, and RT-qPCR and Western blot were used to evaluate the METTL1 expression.

### Immunofluorescence Staining

The gastrocnemius tissues were collected and fixed in 4% paraformaldehyde (#WH1013, Biotechwell) and then dehydrated in 30% sucrose post-hind-limb ischemia. After that, samples were embedded with optimal cutting temperature (OCT) compound and sectioned. Sections were permeated with 0.5% TritonX-100 (#WF1093, Biotechwell) and blocked with 5% BSA (#WH2044, Biotechwell) at room temperature for 1 h and then incubated with pre-diluted CD31 (1:200 dilution, AF3628, R&D System) and alpha-smooth muscle actin (α-SMA) (1:200 dilution, #A5228, Sigma) antibodies at 4°C overnight. Next, the sections were incubated with Alexa Fluor 488 (1:500 dilution, #4412S, CST) and Alexa Fluor 555 antibodies (1:500 dilution, #4413S, CST) at room temperature for 1 h, and the nuclei were stained with DAPI (#WH1163, Biotechwell). Images were visualized in a fluorescence microscope (Leica Microsystems CMS GmbH, Leica Inc., Germany).

### LC-MS/MS

Total RNA was extracted with TRIzol reagent (#15,596,026, Invitrogen), and mRNA was extracted by polyA^+^ purification with Dynabeads mRNA Purification kit (#61006, Invitrogen) according to the manufacturer’s instruction. The mRNA concentration was measured, and about 200–300 ng mRNA was digested with nuclease S1 (#EN0321, Thermo-Fisher) and phosphodiesterase I (#P3141, Sigma-Aldrich). Then, 1 mL of shrimp alkaline phosphatase (rSAP, NEB) was added along with 2.5 mL of 10X CutSmart buffer (NEB) and incubated at 37°C for 2 h. After the incubation, the sample was diluted and filtered with 0.22 mm filters (Millipore). After that, 8 ml of the entire solution was injected into LC-MS/MS. Nucleosides were separated on a C1 column with on-line mass spectrometry detection by an Agilent triple-quadrupole LC mass spectrometer. The nucleosides were quantified with retention time and the nucleoside-to-base ion mass transition of 298.1–166.1 (m7G). Quantitative analysis was performed compared to the standard curve, obtained from pure nucleoside standards running. The m7G level was calculated as the ratio of m7G to G based on calibrated concentration curves.

### RT-qPCR

Total RNA was extracted, and 1 μg total RNA was reverse-transcribed with PrimeScript RT reagent kit (#RR036A, TaKaRa). The cDNA was then amplified with SYBR Green dye (#RR820A, TaKaRa) on a CFX96 Real-Time PCR System (Bio-Rad, Inc., CA, United States). A total reaction system of 10 μL was used, which included 5 μL SYBR Green dye, 1 μL template DNA, 0.5 μL forward primers, 0.5 μL reverse primers, and 3 μL ddH_2_O. The two-step PCR amplification condition was performed: 30 s at 95°C, 5 s at 95°C, and 30 s at 60°C for 39 cycles. β-actin was used as a loading control and relative gene expression was calculated with the standard 2^–△△Ct^ method. The primer sequences are listed in Supplementary Table 1.

### Western Blot

Tissue or HUVEC protein extracts were prepared with lysis buffer, separated by SDS-PAGE, and then transferred to PVDF membranes. The membranes were blocked with 5% BSA and individually incubated with primary antibodies at 4°C overnight. After subsequent washes with TBST, the membranes were then incubated with HRP-linked secondary antibodies at room temperature for 1 h. Blots were scanned using the ChemiDoc Imaging System (Bio-Rad, CA, United States). The intensity was determined with the NIH ImageJ software (1.50i, United States). Antibodies used in this study for Western blot are listed as follows: Mouse anti-METTL1 (1:1,000 dilution, #11525-MM05, Sino Biological), Rabbit anti-METTL1 (1:1,000 dilution, #ab157097, Sino Biological), Mouse anti-VEGFA (1:500, ab1316, Abcam), Goat anti-mouse lgG-HRP (1:3,000 dilution, sc-2005; Santa Cruz), Goat anti-rabbit lgG-HRP (1:5,000, #7074, CST), HRP-linked mouse anti-β-Actin (1:4,000 dilution, #ab20272, Abcam).

### HUVECs Culture and Treatment

Human umbilical vein endothelial cells (HUVECs) were obtained from Fudan IBS Cell Center (Shanghai, China) and cultured in DMEM (#1791920, Gibco) with 10% FBS (#04-001-1A, BI) and 1% penicillin/streptomycin (#10378016, Gibico) in an incubator at 37°C with 5% CO_2_ condition. For hypoxia treatment, HUVECs were placed in a 37°C incubator containing 1% O_2_, 5% CO_2_, and 94% N_2_ condition without FBS for indicated times. HUVECs were transfected with the control (OE-Ctrl) and overexpression METTL1 vectors (OE-METTL1) (Hanbio, Wuhan, China) using Lipofectamine RNAiMAX Transfection Reagent (#13778075, Thermo-Fisher) according to the manufacturer’s protocol. Twenty-four hours after transfection, RT-qPCR and Western blot were used to detect the overexpression efficacy.

### CCK-8 and EdU Assays

CCK-8 (#WH1199, Biotechwel) and EdU (#C10310-1, RiboBio) assays were performed according to the manufacturers’ instructions. Briefly, HUVECs were seeded into a 96-well plate (5,000 cells/well) for the indicated time. After that, 10 μL CCK-8 staining solution per well was added and incubated for another 1 h at 37°C. The absorbance value was detected at 450 nm by a microplate reader (Synergy H4; BioTek Instruments, Inc., United States). For the EdU assay, HUVECs were incubated with EdU solution (50 μM) for 2 h, fixed with 4% paraformaldehyde, decolorized with 2 mg/mL glycine, permeabilized with 0.5% TritonX-100 in PBS), and washed with PBS three times. Then, HUVECs were treated with 100 μL 1 × Apollo Reaction Cocktail for another 30 min. The HUVECs DNA was stained with 100 μL 1 × Hoechst 33342 (1:100 dilution) for 30 min and was visualized in a fluorescence microscope (Leica Microsystems CMS GmbH, Leica Inc., Germany).

### Scratch and Transwell Assay

HUVECs were seeded into 6-well plates. When the HUVECs reached monolayers at ∼70 to 80% confluence, scrape wounds were made in each well with a sterile 10 μL pipette tip. Then, HUVECs were washed with 0.01 M PBS three times to remove cell debris and cultured in the indicated condition. The cells were photographed at the indicated time points. The scratch area was quantitatively evaluated using ImageJ software and normalized to the control group. The migration assay was performed with a 24-well Boyden chamber with porous polycarbonate membrane inserts (8-μm, Corning, NY, United States). In brief, HUVECs (10,000 cells/well) with different treatments were suspended in DMEM supplemented with 0.1% FBS (200 μL) in the upper chamber. The lower chamber was filled with 500 μL DMEM supplemented with 10% FBS. HUVECs that migrated across the filter were washed, stained (0.1% crystal violet), fixed (4% paraformaldehyde), and photographed. Finally, the number of migratory cells was calculated and normalized to the control group.

### Tube Formation Assay

10,000 HUVECs were resuspended in 50 μL endothelial cell growth medium (PromoCell, #C-22010) and seeded onto a 10 μL angiogenesis slide (Ibidi, #81506) pre-coated with polymerization growth factor-reduced Matrigel (BD Biosciences, United Kingdom, #356231). After 6 h of incubation at 37°C, 50 μL calcein (Invitrogen, #2049068) diluted in serum-free medium (1:160 dilution) was added and incubated for another 30 min at room temperature in the dark. Then, calcein was removed by washing three times with PBS. Images were taken with a fluorescence microscope (Leica Microsystems CMS GmbH, Leica Inc., Germany), and tube formation was measured by the Angiogenesis Analyzer using NIH ImageJ software. Relative tube formation was normalized to the control group.

### MeRIP-qPCR

RNA Immunoprecipitation (RIP) followed RT-qPCR (MeRIP-qPCR) were performed using Magna RIP RNA Binding Protein Immunoprecipitation Kit (Millipore, Bedford, MA, United States) according to the manufacturer’s instructions. Briefly, 200 μg of total RNAs were extracted from HUVECs transfected with control (OE-Ctrl) or overexpression plasmid (OE-METTL1) upon hypoxia. Chemically fragmented RNA (∼200 nucleotides) was incubated with mouse Anti-m7G antibody (#RN017M, MBL) or mouse IgG-linked beads in 1 × IP buffer at 4°C overnight. Methylated RNA was immunoprecipitated with beads, eluted by competition with free m7G, and recovered with RNeasy kit (Qiagen). Enrichment of m7G modified VEGFA mRNA in each sample was analyzed by RT-qPCR and calculated by normalizing to input 10-fold. The sequences of the VEGFA mRNA primer for MeRIP-qPCR are listed in Supplementary Table 1.

### ELISA Assay

The concentrations of VEGFA in the supernatants of cultured HUVECs were determined by ELISA using the Human VEGFA ELISA kits (#ab119566, Abcam) according to the manufacturer’s instructions. A standard linear curve of VEGFA concentration derived from the pure VEGFA provided standard solution was generated (*R*^2^ > 0.9). The absorbance value (OD) was detected at 450 nm by a microplate reader (Synergy H4; BioTek Instruments, Inc., United States).

### Statistical Analysis

All data are presented as the mean ± SD. The two-tailed, unpaired Student’s *t*-test was used to determine statistical significance for comparisons between two groups. One-way ANOVA followed by Bonferroni’s *post hoc* test was used to determine statistical significance for comparisons among multiple groups. All experiments were performed as biological replicates (*n* = 3–6) and mentioned in the figure legends. A *P* < 0.05 was considered to be statistically significant. Statistical analysis was performed using GraphPad Prism 8.0 (GraphPad Prism Software Inc., San Diego, CA, United States).

## Results

### m7G and METTL1 Are Decreased in Ischemic Gastrocnemius

**FIGURE 1:**
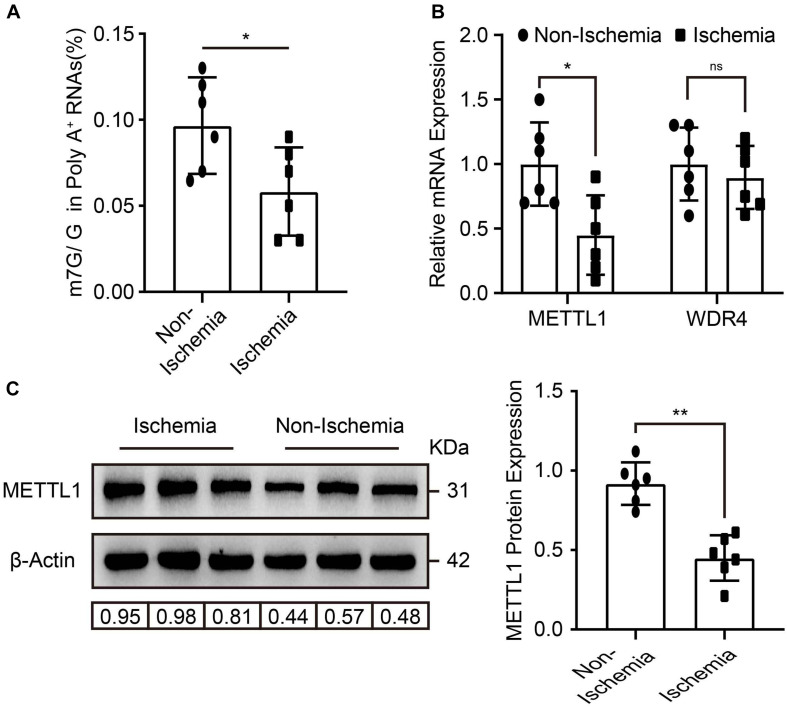
m7G and METTL1 are decreased in ischemic gastrocnemius. **(A)** Quantitative LC-MS/MS analysis of m7G/G of mRNA from the tissues post-ischemia. **(B)** Quantification of *METTL1* and *WDR4* mRNA expression and β-actin was used as a loading control. **(C)** Representative blot image (left panel) and quantitative analysis (right panel) of METTL1 protein expression upon ischemia or non-ischemia. β-actin was used as a loading control. *N* = 6 and all data are presented as the mean ± SD. *P < 0.05; **P < 0.01 and ns indicate no significance.

To explore whether m7G and its enzymes were involved in the regulation of post-ischemic angiogenesis. Mice hind-limb ischemia was constructed. We analyzed the mRNA m7G levels, METTL1, and WDR4 expression in gastrocnemius tissue 3 days post-ischemia. Compared with non-ischemic tissues, LC-MS/MS detected a significant downregulation of m7G post-ischemia (Figure 1A), accompanied by a significant decrease of *METTL1* mRNA expression, whereas an unchanged *WDR4* mRNA expression (Figure 1B). The results were further demonstrated by METTL1 protein expression (Figure 1C). Taken together, these data indicated that the decrease of m7G is mainly associated with METTL1 downregulation in a complicated ischemic condition and suggest that METTL1 may play a role in post-ischemic angiogenesis.

### Improved Blood Flow Recovery and Increased Angiogenesis With Enhanced m7G Upon METTL1 Overexpression Post-ischemic Injury

**FIGURE 2:**
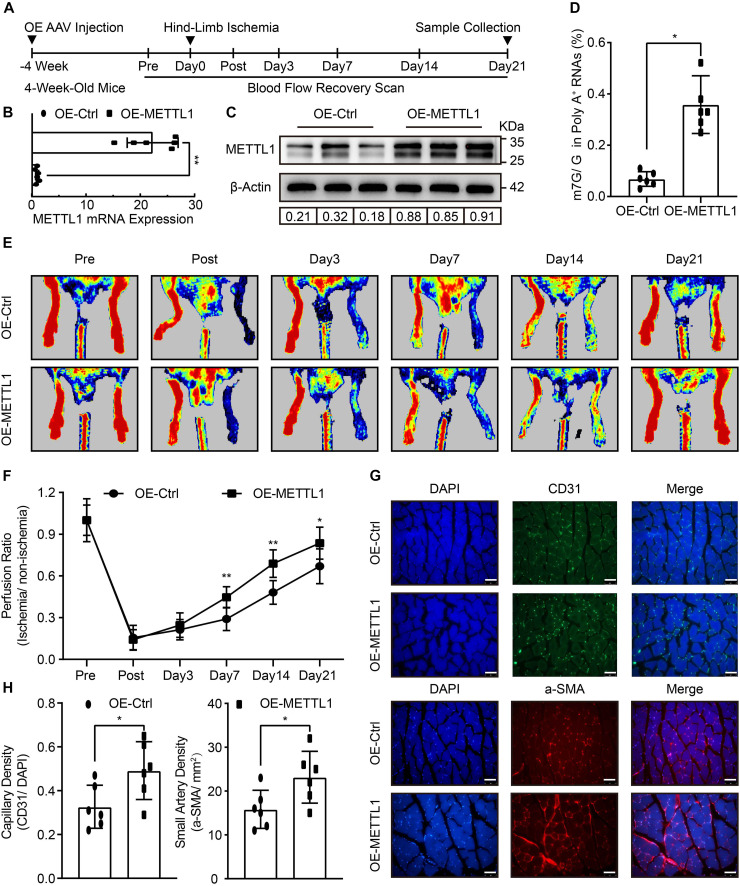
Improved blood flow recovery and increased angiogenesis with enhanced m7G upon METTL1 overexpression post-ischemic injury. **(A)** Schematic illustration of hind-limb ischemia design with METTL1 overexpression. The blood flow recovery was scanned at indicated time points. Twenty-one days post-ischemia, samples were collected for subsequent experiments. **(B)** Quantification of RT-qPCR data for *METTL1* mRNA expression. β-actin was used as a loading control. **(C)** Representative blot image showing METTL1 protein expression. Protein expression was normalized to β-actin. **(D)** Quantitative LC-MS/MS analysis of mRNA m7G levels. **(E)** Representative Laser Doppler images of blood flow recovery scan. **(F)** Quantitative analysis of blood flow recovery data. **(G)** Representative frozen section immunofluorescence images of the angiogenesis markers CD31 and α-SMA expression. **(H)** Quantitative analysis of capillary density (CD31/DAPI) and small artery density (α-SMA/mm^2^). *N* = 6 and all data are presented as the mean ± SD. *P < 0.05; **P < 0.01 and ns indicate no significance compared to the OE-Ctrl.

To determine the role of METTL1 mediated m7G in post-ischemic angiogenesis *in vivo*. The empty AAV (OE-Ctrl) and overexpression AAV (OE-METTL1) were utilized for sustained overexpression of *METTL1* gene by local injection into the gastrocnemius tissues pre-hind-limb ischemia for 4 weeks. After that, hind-limb ischemia was conducted, and blood flow recovery was scanned at indicated time points. Twenty-one days post-ischemia, samples were collected to evaluate METTL1 overexpression efficacy and angiogenic phenotypes (Figure 2A). At 21 days post-ischemia, the results observed a significant METTL1 upregulation of both mRNA level and (Figure 2B) and protein level (Figure 2C and Supplementary Figure 1A). The effect of overexpressed METTL1 on mRNA m7G was further examined by LC-MS/MS. The data revealed a significant increase of mRNA m7G post-ischemia insult (Figure 2D). In addition, the Laser Doppler showed significantly enhanced blood flow recovery (Figures 2E,F) and accompanied by an increased capillary density (CD31 positive) as well as increased small artery density (a-SMA positive) upon METTL1 overexpression post-ischemia (Figures 2G,H).

### Increased HUVECs Angiogenesis With Enhanced m7G Upon METTL1 Overexpression Post-hypoxic Injury

**FIGURE 3:**
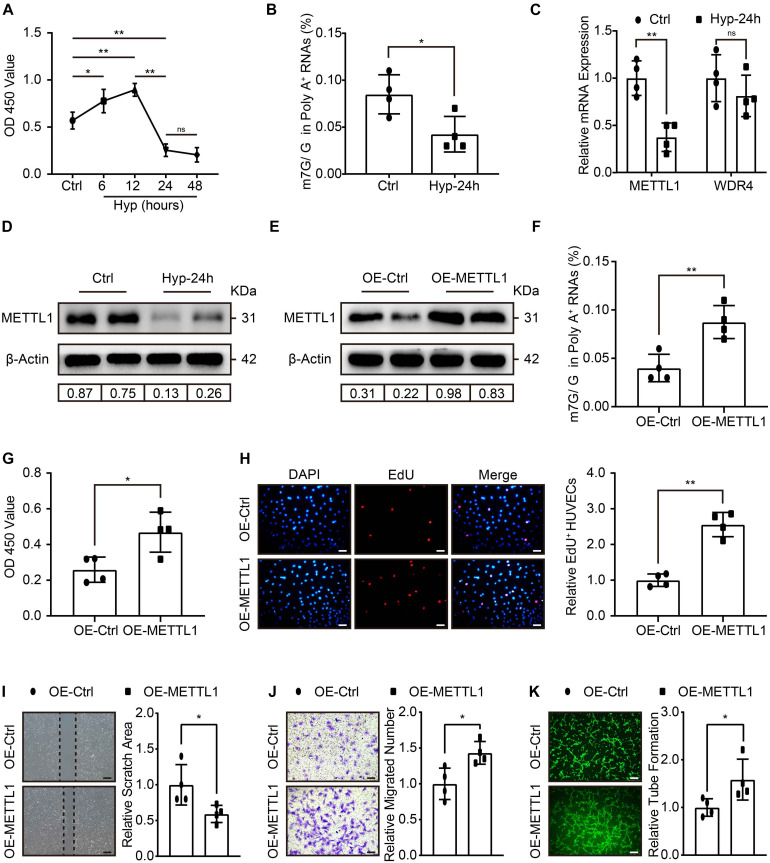
Increased HUVECs angiogenesis with enhanced m7G upon METTL1 overexpression post-hypoxic injury. **(A)** Quantification of HUVECs viability in different hypoxic time points without FBS supplemented in the culture medium. **(B)** Quantitative LC-MS/MS analysis of HUVECs mRNA m7G post-hypoxia. **(C)** Quantification of RT-qPCR data for HUVECs *METTL1* and *WDR4* mRNA expression post-hypoxia. **(D)** Representative blot image showing METTL1 protein expression post-hypoxia. **(E)** Representative blot image showing METTL1 protein overexpression efficacy post-hypoxia. **(F)** Quantitative LC-MS/MS analysis of mRNA m7G levels upon METTL1 overexpression post-hypoxia. **(G)** Quantification of HUVECs viability upon METTL1 overexpression post-hypoxia. **(H)** Representative EdU staining (left panel) and quantitative analysis (right panel) of relative EdU positive HUVECs proportion (EdU^+^/DAPI) upon METTL1 overexpression post-hypoxia. **(I)** Representative scratch closure images (left panel) and quantitative analysis (right panel) of relative scratch area upon METTL1 overexpression post-hypoxia. **(J)** Representative transwell images (left panel) and quantitative analysis (right panel) of relative migrated HUVECs numbers upon METTL1 overexpression post-hypoxia. **(K)** Representative tube formation images (left panel) and quantitative analysis (right panel) of relative tube formation upon METTL1 overexpression post-hypoxia. All experiments were from 4 independent replicates, and data are presented as the mean ± SD. *P < 0.05; **P < 0.01 and ns indicate no significance.

To further explore whether METTL1-mediated m7G could promote angiogenesis *in vitro*. HUVECs were cultured, and the effect of different hypoxic times without FBS supplement on cell viability was observed using the CCK-8 assay. The data showed increased HUVECs viability at 6 and 12 h post-hypoxia compared with the control. Peak viability was observed at 12 h, and a significant decrease occurred at 24 h post-hypoxia. The data further suggest that 48 h post-hypoxia did not further impair HUVECs viability (Figure 3A). Thus, we developed a hypoxia-induced injury model to HUVECs for 24 h. LC-MS/MS was subsequently used, and the results showed that m7G modification underwent significant downregulation post-hypoxic injury (Figure 3B). The expression of METTL1 and WDR4 involved in m7G modifications were further examined. Similar to the *in vivo* data, METTL1 but not WDR4 was significantly decreased post-hypoxic injury (Figures 3C,D and Supplementary Figure 1B). To investigate the function of METTL1, plasmids were used to overexpress METTL1, and the angiogenic phenotypes were further observed. The RT-qPCR (Supplementary Figure 1C) and Western blot results (Figure 3E and Supplementary Figure 1D) showed that METTL1 was significantly upregulated after transfected for 24 h of hypoxic condition. Further, overexpressed METTL1 was accompanied by upregulated mRNA m7G (Figure 3F), enhanced cell viability (Figure 3G), increased proportion of EdU-positive cells (Figure 3H), reduced cell scratch area (Figure 3I), increased migrated cell numbers (Figure 3J) and enhanced tube formation (Figure 3K).

### METTL1 Promoted HUVECs Angiogenesis via Increasing VEGFA Translation

**FIGURE 4:**
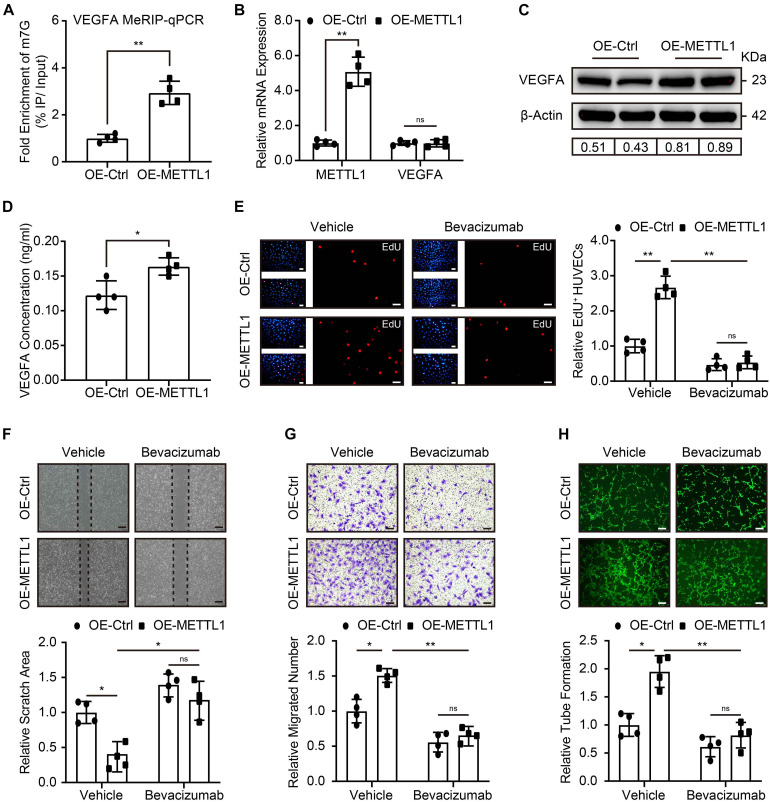
METTL1 promoted HUVECs angiogenesis via increasing VEGFA translation. **(A)** Quantification of MeRIP-qPCR shows the m7G fold enrichment of VEGFA mRNA. **(B)** Quantification of relative *METTL1* and *VEGFA* mRNA expression. **(C)** Representative Western blot images of VEGFA protein expression. **(D)** Quantification of VEGFA concentration in the supernatant of HUVECs culture media. **(E)** Representative EdU staining images (left panel) and quantitative analysis (right panel) of EdU positive HUVECs proportion. **(F)** Representative scratch closure images (upper panel) and quantitative analysis (lower panel) of relative scratch area alteration. **(G)** Representative transwell images (upper panel) and quantitative analysis (lower panel) of relative migrated HUVECs numbers. **(H)** Representative tube formation images (upper panel) and quantitative analysis (lower panel) of relative tube formation. All experiments were from 4 independent replicates, and data are presented as the mean ± SD. *P < 0.05; **P < 0.01 and ns indicate no significance.

Next, we addressed the molecular mechanism that regulates the pro-angiogenic ability of METTL1 mediated m7G in HUVECs. Considering VEGFA signal is crucial for angiogenesis, and m7G is recently reported to promote mRNA translation. Therefore, we first searched in the GEO database for the presence of m7G on VEGFA mRNA. Intriguingly, the m7G sequence data from the GES112276 dataset revealed that m7G modification does exist on VEGFA mRNA. We displayed it using an integrated genome browser (Supplementary Figure 2A). To further validate the results, MeRIP-qPCR was used to detect the VEGFA mRNA m7G alteration. The results showed an increased m7G modification of VEGFA mRNA upon METTL1 overexpression (Figure 4A). Subsequently, we explored the effect of METTL1 overexpression on VEGFA expression. RT-qPCR results indicated that VEGFA mRNA expression did not alter upon forced METTL1 expression (Figure 4B). However, the Western blot results showed an upregulated endogenous VEGFA protein expression (Figure 4C and Supplementary Figure 2B). The ELISA assay detected an enhanced secretion of VEGFA in the supernatant of HUVECs culture media (Figure 4D). To further explore whether the function of METTL1 is dependent on VEGFA expression. One micrometer of Bevacizumab, a humanized monoclonal antibody, specifically binds to all VEGFA with high affinity and inhibits its interaction with VEGFR-1 and VEGFR-2 (Di Mauro et al., 2017; Tan et al., 2017), was used to observe whether the pro-angiogenic function of METTL1 overexpression upon hypoxia was reversed. Compared to the OE-METTL1 group, a decreased cell viability (Supplementary Figure 2C) and relative EdU positive cell proportion (Figure 4E) were observed and indicated a blockade of HUVECs proliferation by Bevacizumab. Next, the increase of scratch area (Figure 4F) and decrease of transwell (Figure 4G) suggested that Bevacizumab treatment blocked the migratory ability promoted by METTL1 overexpression. Finally, the tube formation promoted by forced METTL1 expression was also reserved by Bevacizumab (Figure 4H).

## Discussion

In this study, our results reveal a critical role of m7G in promoting post-ischemic angiogenesis. Present evidence hint that METTL1 is a crucial candidate involves in m7G modification upon ischemic insult, which significantly decreases in a complicated ischemic environment. Forced METTL1 expression promotes mice post-ischemic angiogenesis *in vivo* and increases HUVECs angiogenesis *in vitro* with upregulated mRNA m7G methylation. Mechanically, METTL1 promotes angiogenesis via the increasing of VEGFA translation in an m7G post-transcriptional dependent manner. To the best of our knowledge, this is the first report on the association and mechanism between m7G and post-ischemic disorders.

m7G was reported to participate in tRNA (Cartlidge et al., 2005; Sprinzl and Vassilenko, 2005; Guy and Phizicky, 2014; Lin et al., 2018), rRNA (Motorin and Helm, 2011; Létoquart et al., 2014), and 5′ cap of mRNA (Shatkin, 1976; Furuichi, 2015) modifications. Recently, it has been shown to exist in internal mRNA and plays functional roles (Lin et al., 2018; Zhang et al., 2019). The m7G modification complex, METTL1-WDR4 reported in tRNA and rRNA modification, also installs a subset of internal m7G sites in mRNA (Lin et al., 2018). By extracting and purifying mRNA from post-ischemic tissues and mRNA from HUVECs after hypoxic treating without FBS. LC-MS/MS was applied, and we found a significant decrease in the overall m7G methylation in mRNA. The expression of METTL1 and WDR4 was further examined. The results suggested that only METTL1 expression was decreased while the WDR4 expression did not alter post-ischemia or hypoxic treatment. These data provide side evidence to the previous view that METTL1 is the core mediator in m7G installed by METTL1-WDR4 complex when facing stress conditions (Malbec et al., 2019). We speculate that the expression patterns of METTL1 and WDR4 may differ in various pathologies. However, METTL1 but not WDR4 may be the critical candidate that plays a dominant role for m7G in ischemic disorders, including PAD.

The internal mRNA m7G methylation and its role in ischemic disorders remain unknown. To validate the function of METTL1 mediated m7G in post-ischemic angiogenesis, *in vivo* AAV vectors were used for sustained overexpression, and mice hind-limb ischemia were conducted. Phenotypes of improved blood flow recovery and increased angiogenesis with enhanced m7G methylation in mRNA upon METTL1 overexpression post-ischemia were obtained. Similar to the *in vivo* experiments, *in vitro* overexpression of METTL1 using plasmid resulted in improved proliferation, migration, and tube formation of HUVECs with enhanced m7G methylation in mRNA post-hypoxic injury. These data strongly elucidate the function of METTL1-dependent m7G in mRNA in promoting post-ischemic angiogenesis. Together with m6A in ischemic disorders, our results suggest that m7G and even other types of post-transcriptional RNA modifications may play important functional roles in ischemic cardiovascular diseases, including PAD.

The mRNA m7G methylation has recently been reported to promote mRNA translation efficiency, thus promoting gene expression in a post-transcriptional manner (Zhao et al., 2017; Malbec et al., 2019; Zhang et al., 2019). Whether VEGFA, a widely proven and crucial signal for angiogenesis (Claesson-Welsh and Welsh, 2013; Zou et al., 2019), is involved in METTL1-promoted angiogenesis in an mRNA m7G dependent way remains unknown. To investigate the potential mechanism of METTL1-mediated m7G VEGFA mRNA methylation. m7G MeRIP-seq data from the GEO database were searched, and m7G methylation peak in internal VEGFA mRNA was discovered. According to this peak, primers were designed, and MeRIP-qPCR was performed. The results demonstrate that the m7G methylation of VEGFA mRNA could be upregulated by forced METTL1 expression. Additionally, the VEGFA mRNA expression did not alter but its endogenous protein expression and extracellular secretion were upregulated upon METTL1 overexpression. Moreover, we observed a significant reverse of HUVECs angiogenic phenotypes with the treatment of Bevacizumab, a specific VEGFA inhibitor. This ample evidence demonstrates that METTL1 promotes the translation of VEGFA mRNA by upregulating its m7G and promotes angiogenesis in a VEGFA-dependent manner.

In conclusion, our *in vivo*, *in vitro*, and mechanical evidence uncovered a critical role of METTL1 promoted mRNA m7G methylation in post-ischemic angiogenesis via upregulating VEGFA mRNA translation. These evidence expands our knowledge of RNA epi-transcriptomics in post-ischemic angiogenesis and provides a potential therapeutic option of targeting METTL1 for ischemic cardiovascular diseases, including PAD.

## Limitations

In the present study, the analysis of METTL1 in post-ischemic angiogenesis was achieved using AAV without a specific endothelial promoter. Even though the pro-angiogenic role was demonstrated in cultured HUVECs *in vitro*, it remains to be specified if the enhanced post-ischemic angiogenesis induced by METTL1 upregulation. Specific endothelial genetic engineered approaches are needed for further investigations. Additionally, m7G methylated RNA immunoprecipitation accompanied by next-generation sequencing, polysome and ribosome profiling is required to elucidate the critical mechanism by which METTL1 promotes the translational efficacy of *VEGFA* mRNA or other potential targets. Future studies are warranted to screen more potential and precise candidates in our study setting.

## Data Availability Statement

The original contributions presented in the study are included in the article/Supplementary Material, further inquiries can be directed to the corresponding author/s.

## Ethics Statement

The animal study was reviewed and approved by The Animal Care and Utilization Committee of Fudan University, China.

## Author Contributions

YZ, LK, and ZP conceived, designed, and performed the research. FL and CL participated in part of experiments. XS analyzed the data. BS and JG wrote the manuscript with contributions upon all listed authors. All authors discussed the results, provided comments on this manuscript and agree to be accountable for the content of the work.

## Conflict of Interest

The authors declare that the research was conducted in the absence of any commercial or financial relationships that could be construed as a potential conflict of interest.
