# Supplementary material for: m7G Methyltransferase METTL1 Promotes Post-ischemic Angiogenesis via Promoting VEGFA mRNA Translation
Source: Front Cell Dev Biol. 2021 May 31;9:642080. doi: 10.3389/fcell.2021.642080 (PMC8200671; doi:10.3389/fcell.2021.642080)
Supplement: Supplementary file 1 [file Data_Sheet_1.docx]

Supplementary Materials and Methods

# Supplementary Table 1

# Primers used in this study

| Primers used for RT-qPCR | | | |
| --- | --- | --- | --- |
| Genes | Species | Sequence 5'-3' | |
| METTL1 | Mouse | F | CAGACCACACACTGCGCTA |
|  |  | R | CATCCTTTGGATCATCATGGCTC |
| WDR4 | Mouse | F | AGCCGGTTCCTAGCCTTCT |
|  |  | R | CGTCCTCCCCTTTATCTTCTGG |
| β-actin | Mouse | F | GGCTGTATTCCCCTCCATCG |
|  |  | R | CCAGTTGGTAACAATGCCATGT |
| METTL1 | Human | F | CCGACCCACATTTCAAGCG |
|  |  | R | TCCAGCACATCGGTTATGGTA |
| WDR4 | Human | F | TAACCGATGACAGTAAGCGTCT |
|  |  | R | TCTCCTCCGAGGCTATGAAAG |
| VEGFA | Human | F | AGGGCAGAATCATCACGAAGT |
|  |  | R | AGGGTCTCGATTGGATGGCA |
| β-actin | Human | F | CATGTACGTTGCTATCCAGGC |
|  |  | R | CTCCTTAATGTCACGCACGAT |
| METTL3 | Human | F | TTGTCTCCAACCTTCCGTAGT |
|  |  | R | CCAGATCAGAGAGGTGGTGTAG |
| METTL14 | Human | F | GAACACAGAGCTTAAATCCCCA |
|  |  | R | TGTCAGCTAAACCTACATCCCTG |
| WTAP | Human | F | CTTCCCAAGAAGGTTCGATTGA |
|  |  | R | TCAGACTCTCTTAGGCCAGTTAC |
| METTL16 | Human | F | TTCTGTCAAGGTCGGACAATG |
|  |  | R | CAGCACCACGAATGTTATGGG |
| FTO | Human | F | ACTTGGCTCCCTTATCTGACC |
|  |  | R | TGTGCAGTGTGAGAAAGGCTT |
| ALKBH5 | Human | F | CGGCGAAGGCTACACTTACG |
|  |  | R | CCACCAGCTTTTGGATCACCA |
|  | | | |
| Primers used for MeRIP-qPCR | | | |
| Genes | Species | Sequence 5'-3' | |
| VEGFA | Human | F | CGGGAACCAGATCTCTCACC |
|  |  | R | CCTCCCAACTCAAGTCCACA |

# Supplementary Method

# m6A dot blot

Total RNAs were extracted, and the concentrations were adjusted by serial dilution to 50 ng/μL and 100 ng/μL for one assay. The diluted total RNAs were denatured at 95°C for 3 minutes to disrupt any secondary structures. Then, 2 μL of serially diluted RNAs were dropped onto a Hybond-N^+^ membrane (GE Healthcare, #RPN203B) and cross-linked with a Stratalinker 2400 UV Crosslinker (1,200 µJ [×100]; 25–50 s). The membrane was then washed and blocked with 5% BSA for 1 hour at room temperature and subsequently incubated overnight at 4°C with anti-m6A antibody (S Tab. 1). After extensive washing, the Hybond-N^+^ membrane was incubated with HRP-linked secondary antibody at room temperature for 1 hour and exposed to ECL substrate (Thermo, #32132). The same amount of total RNAs was stained with 0.02% methylene blue (MB) in ddH_2_O for 2 hours at room temperature and then photographed to verify the equal loading of RNA.

# Supplementary Figure 1


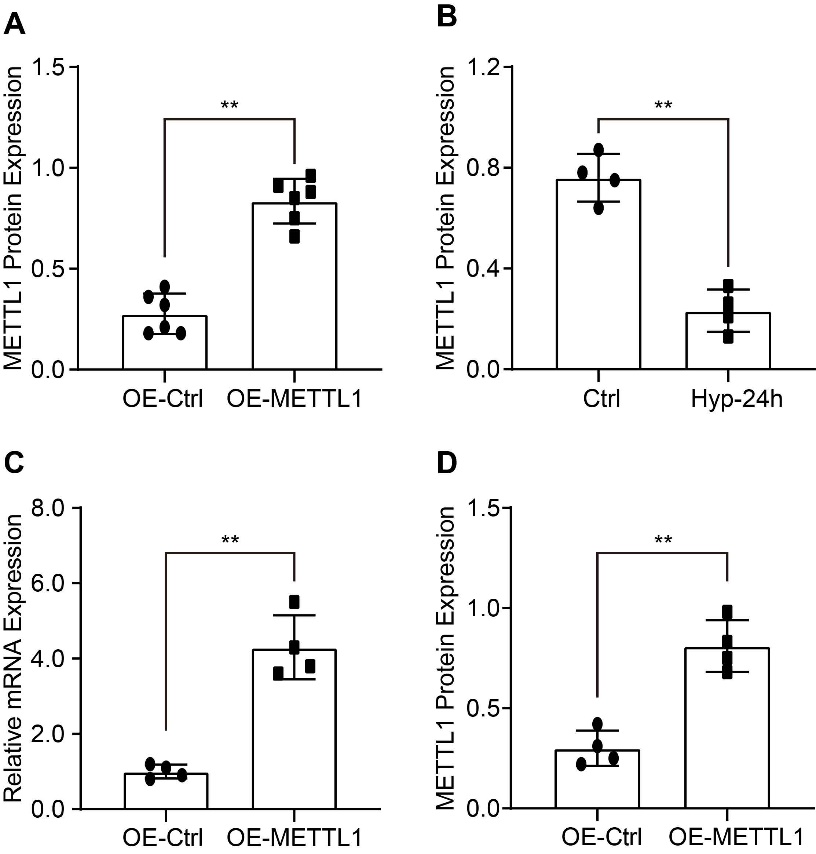


**Supplementary Figure 1.** **A**, Quantitative analysis of AAV mediated gastrocnemius METTL1 protein overexpression efficacy at 21 days post-ischemia (n=6). **B**, Quantitative analysis of HUVECs METTL1 protein expression post-hypoxia for 24 hours supplemented without FBS (n=4). **C**, Quantitative RT-qPCR analysis of *METTL1* mRNA expression after plasmid transfection for 24 hours (n=4). **D**, Quantitative analysis of METTL1 protein overexpression efficacy after plasmid transfection for 24 hours (n=4). Data were presented as the mean ± SD. * *P*<0.05; ** *P*<0.01 and the ns indicate no significance.

# Supplementary Figure 2


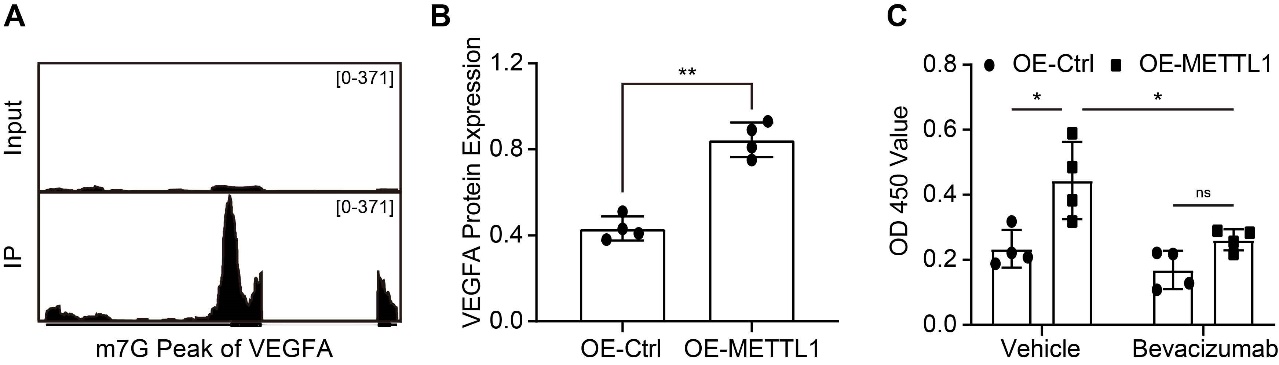


**Supplementary Figure 2. A**, Integrated genome browser views of m7G modification within VEGFA mRNA from the input and IP sequence data from the GEO database (GES112276). **B**, Quantitative analysis of VEGFA protein expression upon METTL1 overexpression in hypoxic condition. **C**, Quantitative analysis of CCK-8 data with the addition of vehicle or 1uM of Bevacizumab upon METTL1 overexpression in hypoxic condition. All data were from 4 independent replicates and presented as the mean ± SD. * *P*<0.05; ** *P*<0.01 and the ns indicate no significance.

# Supplementary Figure 3

#
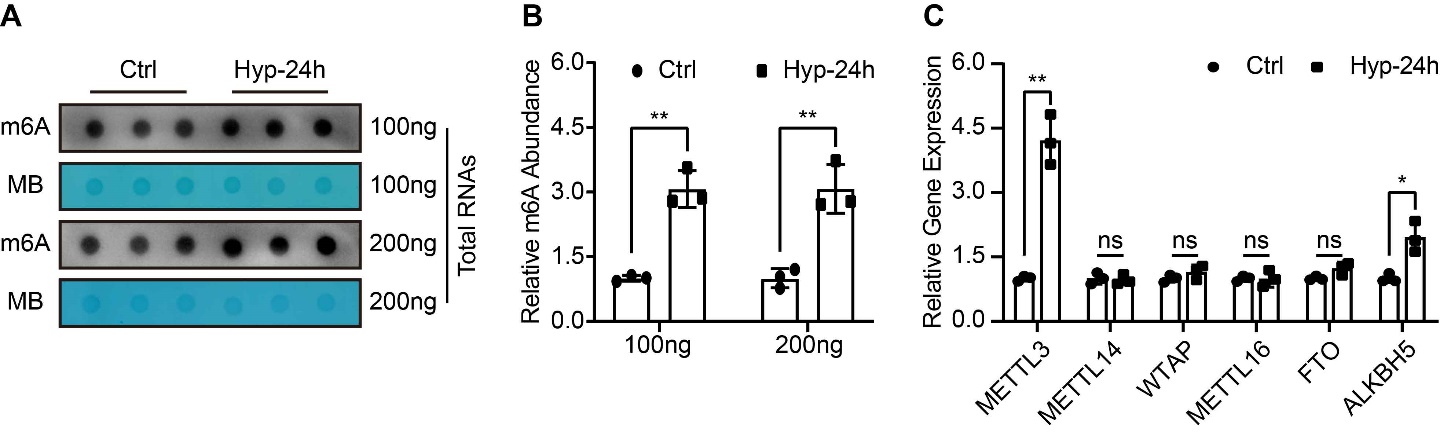


**Supplementary Figure 3.** **A-B**, Dot blot images and quantitative analysis of total RNAs m6A abundance of HUVECs under the normoxic (Ctrl) or hypoxic (Hyp-24h) conditions. The same amount of total RNAs was stained with 0.02% methylene blue (MB) for equal loading. **C**, RT-qPCR quantification of m6A related methyltransferases and demethylases expression. The β-actin was used as a loading control. Data were from 3 independent replicates and presented as the mean ± SD. * *P*<0.05; ** *P*<0.01 and the ns indicate no significance.
